# Crizotinib: A Novel Strategy to Reverse Immunosuppression in Melanoma by Targeting Lactate Transport

**DOI:** 10.1002/mco2.70286

**Published:** 2025-07-21

**Authors:** Zhe Zhou, Xu Zhang, Susi Zhu, Waner Liu, Yeye Guo, Siyu Xiong, Cong Peng, Xiang Chen

**Affiliations:** ^1^ The Department of Dermatology Xiangya Hospital Central South University Changsha China; ^2^ Furong Laboratory Changsha China; ^3^ Hunan Key Laboratory of Skin Cancer and Psoriasis Xiangya Hospital Central South University Changsha China; ^4^ Hunan Engineering Research Center of Skin Health and Disease Xiangya Hospital Central South University Changsha China; ^5^ National Clinical Research Center for Geriatric Disorders Xiangya Hospital Central South University Changsha China

**Keywords:** CD147, CXCL13, lactate, lactylation, monocarboxylate transporter 1

## Abstract

Lactate is a vital metabolite in cancer, significantly impacting tumor progression, metastasis, and overall survival. The CD147–monocarboxylate transporter 1 (MCT1) complex, a major lactate transporter, has emerged as a promising therapeutic target. However, no effective protein–protein interaction (PPI) inhibitors targeting the CD147–MCT1 complex have been identified. In this study, we found that the small‐molecule inhibitor crizotinib effectively disrupts the CD147–MCT1 interaction, leading to reduced lactate secretion from melanoma cells and decreased lactate uptake by macrophages. In vivo studies demonstrated that crizotinib treatment significantly suppressed tumor growth and enhanced responsiveness to immune checkpoint blockade therapy. Flow cytometry revealed that this metabolic intervention inhibits M2 polarization and reshapes the tumor immune microenvironment. Transcriptomic analysis further revealed that lactate induces C‐X‐C motif chemokine ligand 13 (CXCL13) expression in macrophages, which enhances melanoma invasiveness and impairs immune cell‐mediated cytotoxicity. Importantly, crizotinib suppresses CXCL13 expression by blocking lactate‐driven histone lactylation, thereby reversing the transcriptional reprogramming induced by lactate, as evidenced by reduced histone H3 lysine 18 lactylation (H3K18la) enrichment at the CXCL13 promoter. Taken together, these findings provide new insights into targeting metabolic‐immune crosstalk and highlight the value of disrupting CD147–MCT1 interactions to improve immunotherapeutic responses in patients with melanoma.

## Graphical abstract

Crizotinib inhibits CD147‐MCT1‐mediated lactate transport, suppressing melanoma growth and immune escape, prevents M2 macrophage polarization, and enhances immunotherapy by reducing CXCL13 via histone lactylation.

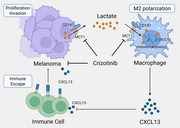

## 1 Introduction

Melanoma is an aggressive skin cancer whose incidence continues to rise globally. A distinguishing feature of this malignancy is its exceptional metabolic adaptability, which allows tumor cells to survive and proliferate across a range of microenvironments, from oxygen‐rich primary lesions to nutrient‐deprived metastatic sites. A central component of this adaptability is the persistent reliance of tumors on aerobic glycolysis [1]. Lactate, the primary by‐product of this pathway, was once considered a metabolic waste product but is now recognized as a bioactive metabolite that orchestrates tumor–immune interactions and drives disease progression. Increasing evidence suggests that this metabolic reprogramming not only sustains rapid tumor proliferation [2, 3] but also fosters an immunosuppressive microenvironment conducive to immune evasion and metastatic dissemination [4, 5, 6].

Tumor‐associated macrophages (TAMs), the most abundant immune cell population within the tumor microenvironment (TME), are particularly sensitive to lactate‐driven signaling. These cells exhibit remarkable phenotypic plasticity, responding to environmental cues with either pro‐ or antitumor activity [7, 8]. Elevated extracellular lactate levels promote macrophage polarization toward the M2 phenotype, a state closely associated with immunosuppression, angiogenesis, and metastatic potential [9, 10]. Moreover, recent studies have indicated that lactate is crucial for immune cell reprogramming. Through a process known as lactylation [11], lactate can modify proteins within macrophages, leading to changes in gene expression that favor an immunosuppressive phenotype [12, 13]. This modification enhances their protumorigenic functions and contributes to the overall immunosuppressive nature of the TME. Thus, targeting the CD147–MCT1 interaction to inhibit lactate transport and suppress M2‐like macrophage differentiation represents a promising therapeutic strategy for melanoma.

Given the central role of lactate in shaping the immunometabolic landscape, the mechanisms by which lactate is exported from melanoma cells have drawn increasing attention. To facilitate glycolysis and prevent intracellular acidification, cancer cells exhibiting high glycolytic activity rely on the active export of lactate, a process primarily mediated by the monocarboxylate transporters MCT1 and MCT4, whose activity is influenced by tissue type and environmental conditions. Typically, MCT1 primarily facilitates lactate uptake, whereas MCT4 is specialized for lactate export. However, in highly glycolytic cells, MCT1 may also assume the role of MCT4, enabling lactate export when necessary [14, 15]. A key distinction between these transporters is their affinity for lactate: MCT1 has a significantly greater affinity, with a Km ranging from 3.5 to 10 mM, than MCT4, whose Km is between 22 and 28 mM [16]. The functionality of these transporters is significantly enhanced by their association with the chaperone protein CD147, a transmembrane glycoprotein implicated in cancer progression and metastasis [17, 18, 19], which stabilizes MCT1/4 on the cell surface and facilitates their lactate transport activity [20, 21].

PPI inhibitors have emerged as a promising class of therapeutic agents due to their enhanced specificity and efficacy and reduced systemic toxicity compared to conventional small‐molecule drugs [22, 23]. By selectively disrupting disease‐related protein complexes, PPI inhibitors offer unique advantages. Despite the well‐established role of the CD147–MCT1 complex in facilitating lactate transport and promoting tumor progression, no PPI inhibitors have been developed to specifically target this interaction. Existing MCT1‐targeted agents, such as AZD3965, primarily inhibit lactate transport but fail to disrupt the CD147–MCT1 regulatory interface, thus limiting their therapeutic precision.

In this study, we revealed that crizotinib, a small‐molecule kinase inhibitor, exhibited superior performance in disrupting this interaction during subsequent immunoprecipitation assays and demonstrated its ability to reduce lactate export, thereby modulating macrophage polarization and impairing CXCL13‐mediated immune escape in melanoma. Our findings highlight a previously underexplored regulatory axis in melanoma metabolism and provide a mechanistic link between lactate transport and immune evasion. Moreover, by interfering with the CD147–MCT1 complex, crizotinib limits the metastatic potential of melanoma cells, suggesting a dual mechanism of action. These results suggest that repurposing PPI inhibitors such as crizotinib to target metabolic‐immune crosstalk may represent a promising therapeutic avenue for treating patients with melanoma, warranting further exploration of combination strategies with immunotherapies.

## 2 Results

### 2.1 TR‐FRET Screening Assay for CD147–MCT1 Interaction Inhibitors

**FIGURE 1:**
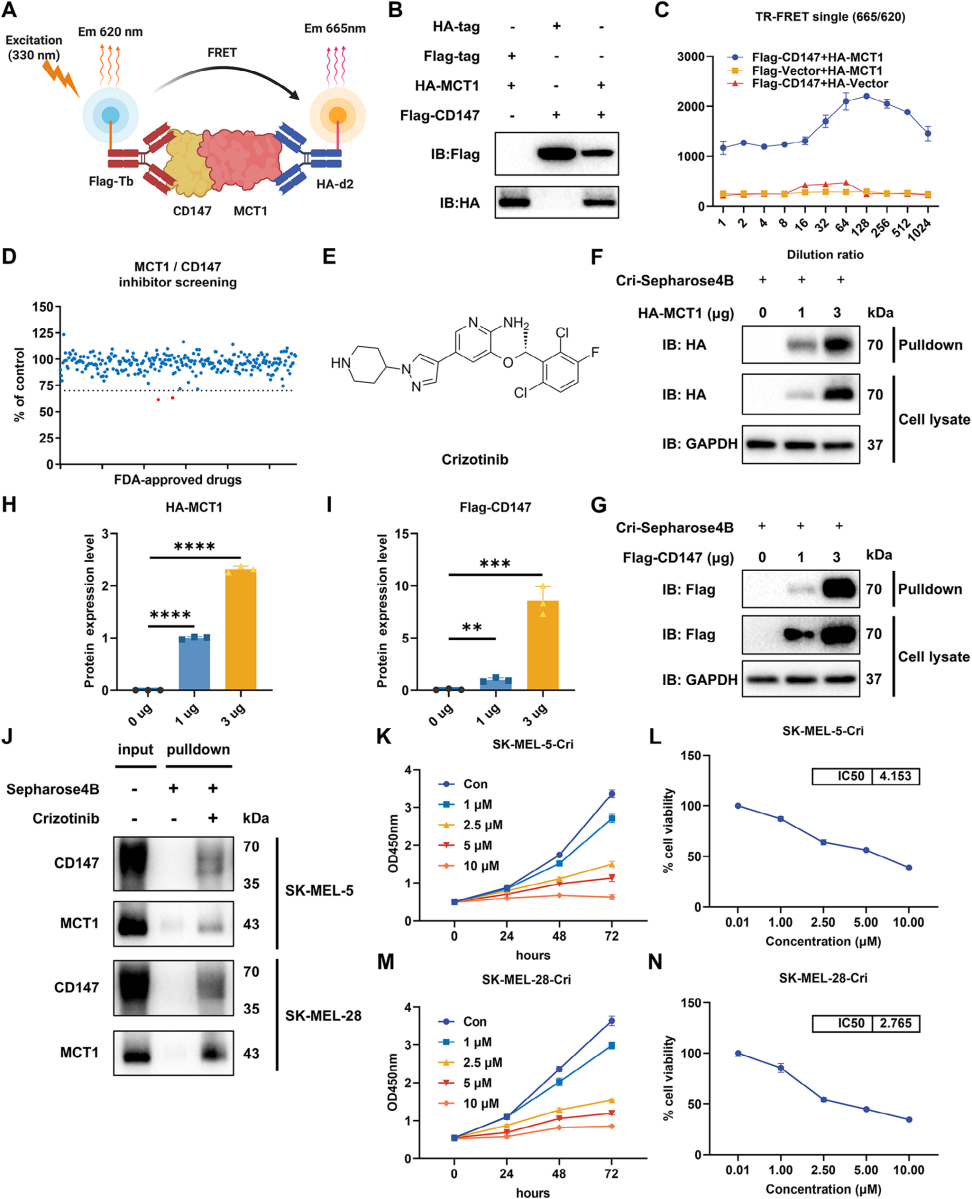
TR‐FRET screening and targeting validation. (A) Schematic representation of the TR‐FRET assay. CD147 and MCT1 were tagged with Flag‐Tb and HA‐d2, respectively. (B) 293T cells were transfected with HA‐MCT1, Flag‐CD147, HA vector, or Flag vector. (C) After substrate addition to the 293T cell lysates and subsequent incubation, signals were recorded at 615 and 665 nm. The *x*‐axis represents the lysate dilution ratio, whereas the *y*‐axis represents the TR‐FRET signal values after background subtraction (*n* = 3). (D and E) High‐throughput screening of FDA‐approved drugs via the MCT1/CD147 TR‐FRET assay. Crizotinib and axitinib are highlighted in red. (E) Chemical structure of crizotinib. (F and G) CRI‐Sepharose 4B (CRI: Crizotinib) was incubated with lysates from 293T cells overexpressing HA‐MCT1 (F) or Flag‐CD147 (G), and WB was used to examine the binding of these proteins to CRI‐Sepharose 4;, CRI represents the crizotinib. (H and I) Cri‐Sepharose 4B binding protein expression of HA‐MCT1 (H) and Flag‐CD147 (I) in response to increasing amounts of transfected plasmids (0, 1, and 3 µg). (J) CRI‐Sepharose 4B or Sepharose 4B was incubated with lysates from SK‐MEL‐5 or SK‐MEL‐28 cells to investigate the interaction of crizotinib with CD147 and MCT1. (K–N) CCK‐8 assays were used to evaluate the impact of crizotinib on SK‐MEL‐5 (K, L) and SK‐MEL‐28 (M, N) cells. The cells were treated with different concentrations of crizotinib (1, 2.5, 5, and 10 µM). The data are presented as the means ± SDs (*n* = 3).

Since the interaction between CD147 and MCT1 plays pivotal roles in metabolic regulation and the microenvironment, we utilized the TR‐FRET technique, which is sensitive, stable, and efficient, to screen specific inhibitors (Figure 1A). CD147 and MCT1 were overexpressed in 293T cells (Figure 1B), and TR‐FRET signals were detected to evaluate their interaction. We used diluted protein at various concentrations to determine the optimal protein concentration for subsequent screening experiments (Figure 1C). We subsequently evaluated 276 selected compounds from the Food and Drug Administration (FDA)‐approved drug library using the TR‐FRET screening system to identify inhibitors capable of effectively disrupting CD147–MCT1 complex formation (Figure 1D). This rigorous screening process led to the identification of two promising candidate compounds, crizotinib (Figure 1E) and axitinib, both of which significantly reduced the TR‐FRET signal, indicating their potential as inhibitors of the CD147–MCT1 interaction. To further validate the inhibitory effects of both compounds, coimmunoprecipitation was performed to assess the CD147–MCT1 interaction in the presence of crizotinib or axitinib. Notably, crizotinib exhibited greater efficacy than did axitinib, substantially blocking the formation of the CD147–MCT1 complex (Figure S1A). To determine whether crizotinib contributes directly to the inhibition of the CD147–MCT1 interaction, pulldown assays were employed, and the interaction of crizotinib with overexpressed CD147 and MCT1 is depicted in Figure 1F,G, confirming its potential as a CD147–MCT1 inhibitor. Quantitative analysis of the pulldown results is shown in Figures 1H,I. Consistent with the results obtained in 293T cells, crizotinib interacted directly with endogenous CD147 and MCT1 in melanoma cells (Figure 1J). Moreover, by performing Cell Counting Kit‐8 (CCK‐8) assays to assess the impact of crizotinib on melanoma cell proliferation, we found that crizotinib significantly inhibited melanoma cell growth (Figure 1K–N). In addition, crizotinib had a significantly lower inhibitory effect on normal cells (Figure S1B), further underscoring its potential as a therapeutic agent. Together, these results indicate that crizotinib is a novel PPI that blocks the CD147–MCT1 interaction.

### 2.2 Crizotinib Inhibits the Invasion and Metastasis of Melanoma and Disrupts the Membrane Localization of MCT1

**FIGURE 2:**
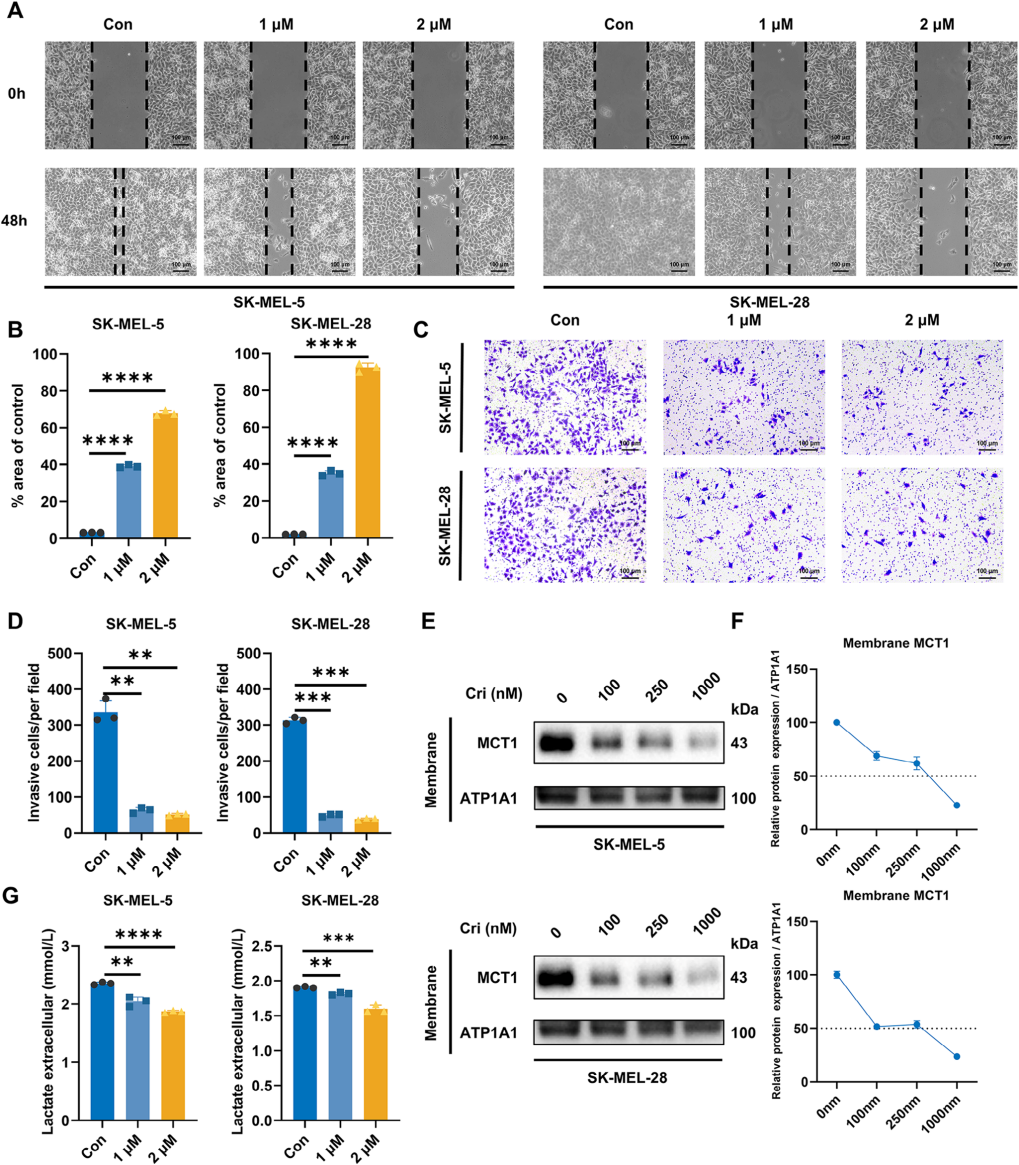
Crizotinib inhibits the proliferation, invasion, and metastasis of melanoma and disrupts the membrane localization of MCT1. (A) Wound healing assays were performed in SK‐MEL‐5 and SK‐MEL‐28 cells to assess the impact of crizotinib on cell migration, with the control (Con) group serving as a baseline (*n* = 3). (B) Quantitative analysis of the wound healing assays. (C) The effect of crizotinib on cell invasion was evaluated via Transwell assays (*n* = 3). (D) Quantitative analysis of the cell invasion results. (E) Membrane proteins were extracted from SK‐MEL‐5 and SK‐MEL‐28 cell pellets following crizotinib treatment. (F) Western blot analysis and quantitative assessment of MCT1 protein levels. (G) Lactate concentrations in the supernatants from crizotinib‐treated SK‐MEL‐5 and SK‐MEL‐28 cell cultures were measured.

Melanoma is a highly aggressive and lethal form of skin cancer, and its initiation and progression are closely associated with invasion and metastasis. To evaluate the antimetastatic potential of crizotinib, both wound healing and Transwell assays were performed. As a result, untreated control cells exhibited significant wound closure after 48 h, whereas crizotinib‐treated cells demonstrated dose‐dependent inhibition of migration (Figure 2A,B). Consistent with these findings, Transwell assays revealed a marked reduction in the number of cells that penetrated the Matrigel‐coated membranes following crizotinib treatment (Figure 2C,D).

Considering the importance of lactate metabolism in tumor progression, we hypothesized that crizotinib could obstruct the CD147–MCT1 interaction and influence MCT1 localization on the cell membrane, thereby affecting lactate transport in melanoma cells. To further confirm our hypothesis, cells were exposed to varying doses of crizotinib, and membrane proteins were isolated and visualized via western blotting, which revealed that MCT1 expression on the cell membrane decreased with increasing crizotinib concentration (Figure 2E,F). Consequently, crizotinib treatment led to a decrease in extracellular lactate levels, which was consistent with the downregulation of MCT1 expression (Figure 2G). In addition, we knocked down CD147 and MCT1 in the cells. This resulted in reduced expression of both proteins on the membrane (Figure S1C,D) and decreased the extracellular concentration of lactate (Figure S1E), which is consistent with the effects noted following crizotinib treatment. These findings demonstrate that crizotinib effectively impairs melanoma cell invasion and metastasis, which may account for the blockade of lactate transport via disruption of MCT1 membrane localization.

### 2.3 Crizotinib Improves the Therapeutic Efficacy of PD‐1 Blockade by Modulating Macrophage Polarization and Promoting CD8⁺ T‐Cell Responses

**FIGURE 3:**
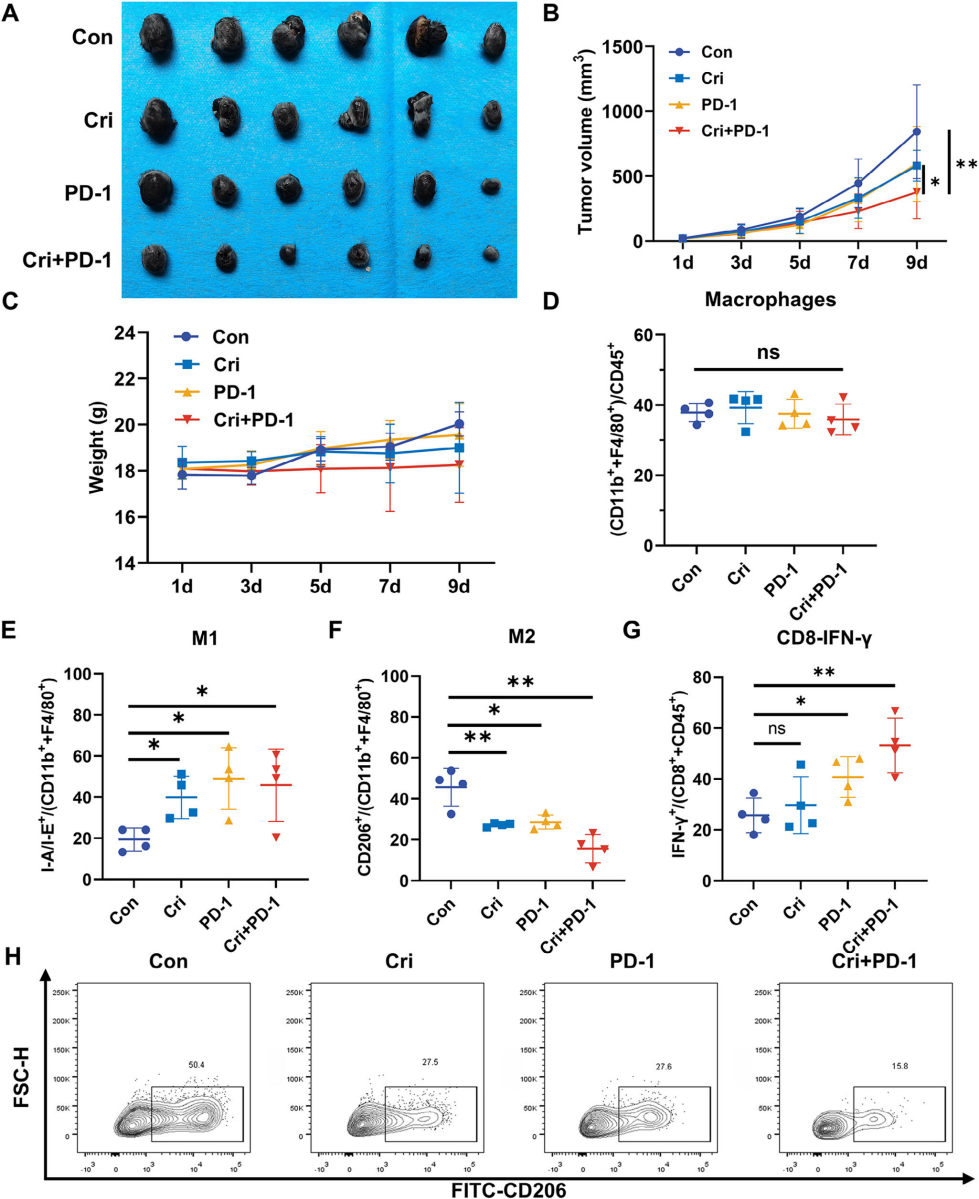
Crizotinib enhances the efficacy of PD‐1 therapy in vivo by inhibiting macrophage polarization toward the M2 phenotype. (A) Cri represents the crizotinib treatment group, while PD‐1 refers to the PD‐1 mAb treatment group. Representative images of tumors collected from mice on Day 9 post‐treatment. Each group included six mice. (B) Tumor volume measurements during treatment; data are presented as the means ± SDs. (C) Mouse body weights were monitored throughout the treatment to assess potential side effects. (D) Quantification of tumor‐infiltrating macrophages (CD11b⁺F4/80⁺) by flow cytometry (*n* = 4 mice per group). (E) Analysis of M1 macrophages (I‐A/I‐E⁺CD11b⁺F4/80⁺) in tumor tissues via flow cytometry. (F) Analysis of M2 macrophages (CD206⁺CD11b⁺F4/80⁺). (G) Percentage of IFN‐γ⁺ CD8⁺ T cells (CD8⁺CD45⁺) among tumor‐infiltrating lymphocytes. (H) Representative flow cytometry plots showing CD206 expression in macrophages from each treatment group.

Lactate, a key metabolic by‐product, is known to impair antitumor immunity [24]. To evaluate the impact of crizotinib on immunotherapy efficacy, we administered an anti‐PD‐1 mAb to immune‐competent mice bearing B16‐F10 melanoma tumors. Our experiments demonstrated significant inhibition of tumor growth with monotherapy via either crizotinib or PD‐1 immune checkpoint blockade (Figure 3A–C). Notably, the combined treatment further decreased the tumor volume and weight, indicating a potential synergistic effect between the two modalities. To explore the downstream mechanism by which crizotinib contributes to immunotherapy, flow cytometry analysis was performed. Interestingly, combined treatment with crizotinib and the anti‐PD‐1 mAb resulted in no significant changes in the overall macrophage population (Figure 3D), whereas substantial alterations in macrophage polarizations were observed, in which the proportion of anti‐inflammatory and antitumor M1 macrophages notably increased (Figure 3E), whereas the subpopulation of M2 macrophages associated with immunosuppression and tumor promotion decreased (Figure 3F,H). Moreover, the proportion of IFN‐γ⁺ CD8⁺ T cells, indicative of enhanced cytotoxic activity, was significantly elevated in the combination group (Figure 3G). This immunomodulatory effect of crizotinib was further validated in the YUMM1.7 melanoma model, where combination treatment similarly reduced M2 macrophage populations and enhanced IFN‐γ⁺ CD8⁺ T‐cell responses (Figure S2). These findings indicate that crizotinib improved the therapeutic efficacy of PD‐1 blockade by modulating macrophage polarization and promoting CD8⁺ T‐cell effector function.

### 2.4 Crizotinib Inhibits Macrophage Lactate Uptake and Promotes M2 Polarization

**FIGURE 4:**
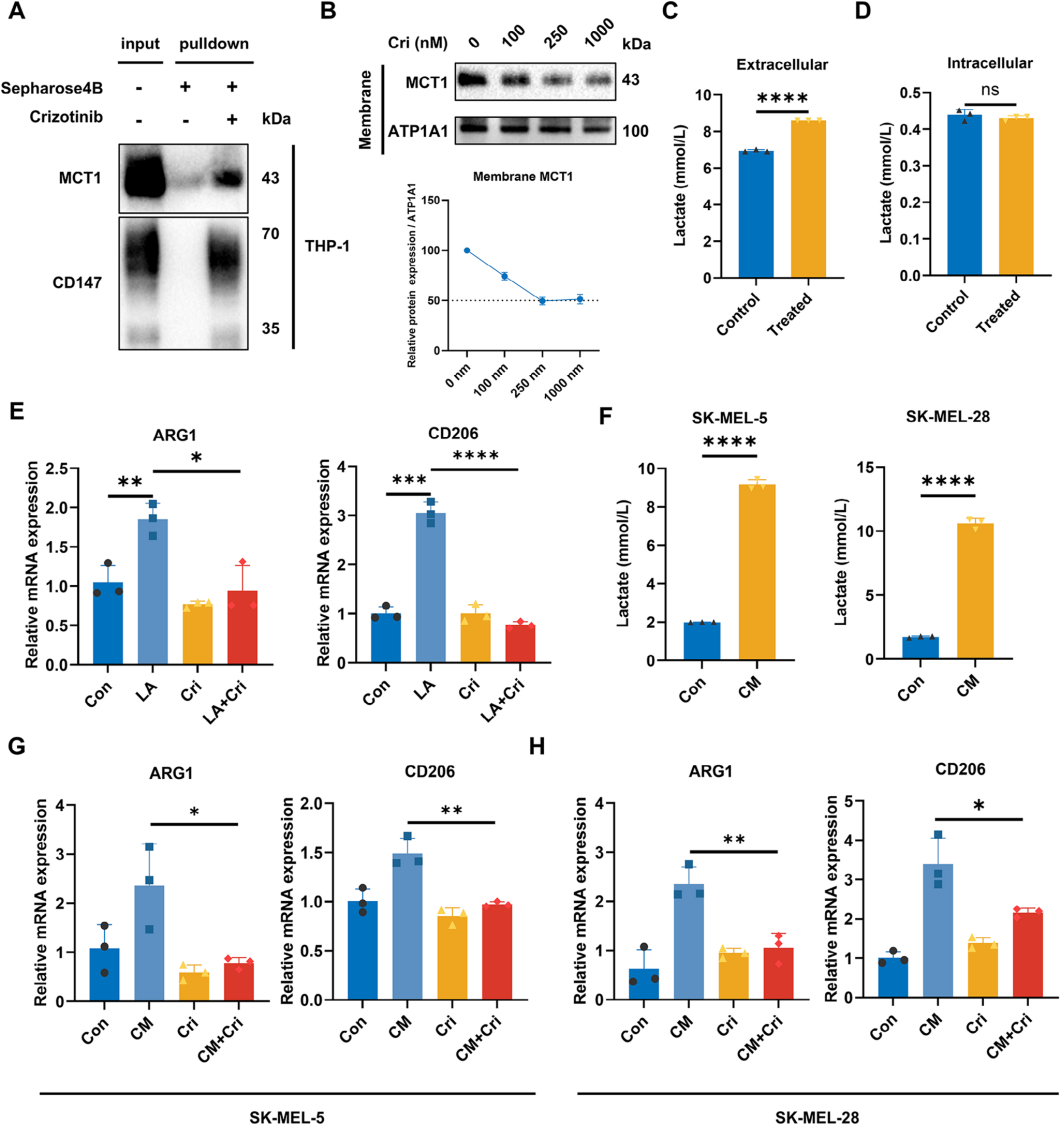
Crizotinib reduces lactate uptake by macrophages and inhibits their differentiation into the M2 phenotype. (A) Pulldown assays were conducted to investigate the interaction of crizotinib with CD147 and MCT1 in macrophages. (B) Membrane proteins were extracted from THP‐1 cells treated with varying concentrations of crizotinib (0–1000 nM) and analyzed via immunoblotting for MCT1 using ATP1A1 as a control. (C and D) Lactate concentrations were measured in the extracellular medium (C) and intracellular compartments (D) after crizotinib treatment to evaluate the effect of crizotinib on lactate uptake (*n* = 3). (E) RT‐PCR was used to assess M1 (ARG1) and M2 (CD206) marker expression in THP‐1 macrophages treated with lactate, with and without crizotinib. The data are presented as the means ± SDs (*n* = 3). The LA group represents the lactate treatment group. (F) Conditioned culture media from tumor cells were collected, and the lactate concentration in the supernatant was measured. (G and H) Macrophages were treated with the CM, and the transcriptional changes in M1/M2 markers were analyzed to evaluate the impact of the CM on macrophage polarization. The data are presented as the means ± SDs (*n* = 3).

Although crizotinib affects lactate secretion in melanoma cells, several studies have suggested that MCT1 mediates lactate transport in a concentration gradient‐dependent manner. Given the potential differences in lactate transport direction between macrophages and melanoma cells, we investigated the effects of crizotinib on lactate uptake and polarization in macrophages. Pull‐down assays confirmed the interaction of crizotinib with CD147 and MCT1 in macrophages (Figure 4A). To assess the effect of crizotinib on MCT1 expression, membrane proteins were extracted from THP‐1 cells treated with varying concentrations of crizotinib (0–1000 nM). Immunoblotting of the membrane fractions revealed a dose‐dependent decrease in MCT1 expression (Figure 4B). We subsequently treated THP‐1 cells with a medium containing 10 mM lactate and assessed changes in the lactate concentration to evaluate the influence of crizotinib on lactate transport. The results revealed that the lactate concentration in the control group was approximately 6.93 mM, whereas the lactate concentration in the crizotinib‐treated macrophages was significantly higher, at approximately 8.60 mM (Figure 4C), suggesting that lactate uptake by crizotinib‐treated THP‐1 cells was inhibited. There was no significant change in the intracellular lactate concentration (Figure 4D), possibly because lactate is absorbed and subsequently used for synthesis or metabolism, thus maintaining intracellular homeostasis and preventing excessive accumulation of lactate.

Extensive research has demonstrated that lactate facilitates the M2 polarization of macrophages [6, 9, 10]. Therefore, we investigated the impact of lactate and crizotinib on macrophage polarization. Consistent with previous findings, lactate treatment significantly increased the expression of arginase 1 (ARG1) and CD206, whereas cotreatment with crizotinib markedly reduced the expression of M2 polarization markers (Figure 4E). Similar trends were observed when macrophages were treated with conditioned media (CM) collected from tumor cells, which contain higher lactate concentrations (Figure 4F–H). Additionally, we observed comparable results in mouse macrophage cell lines (Figure S3). Together, these results demonstrated that crizotinib inhibited M2 polarization of macrophages by hindering lactate uptake.

### 2.5 Lactate Promotes M2 Polarization of Macrophages and Increases CXCL13 Secretion

**FIGURE 5:**
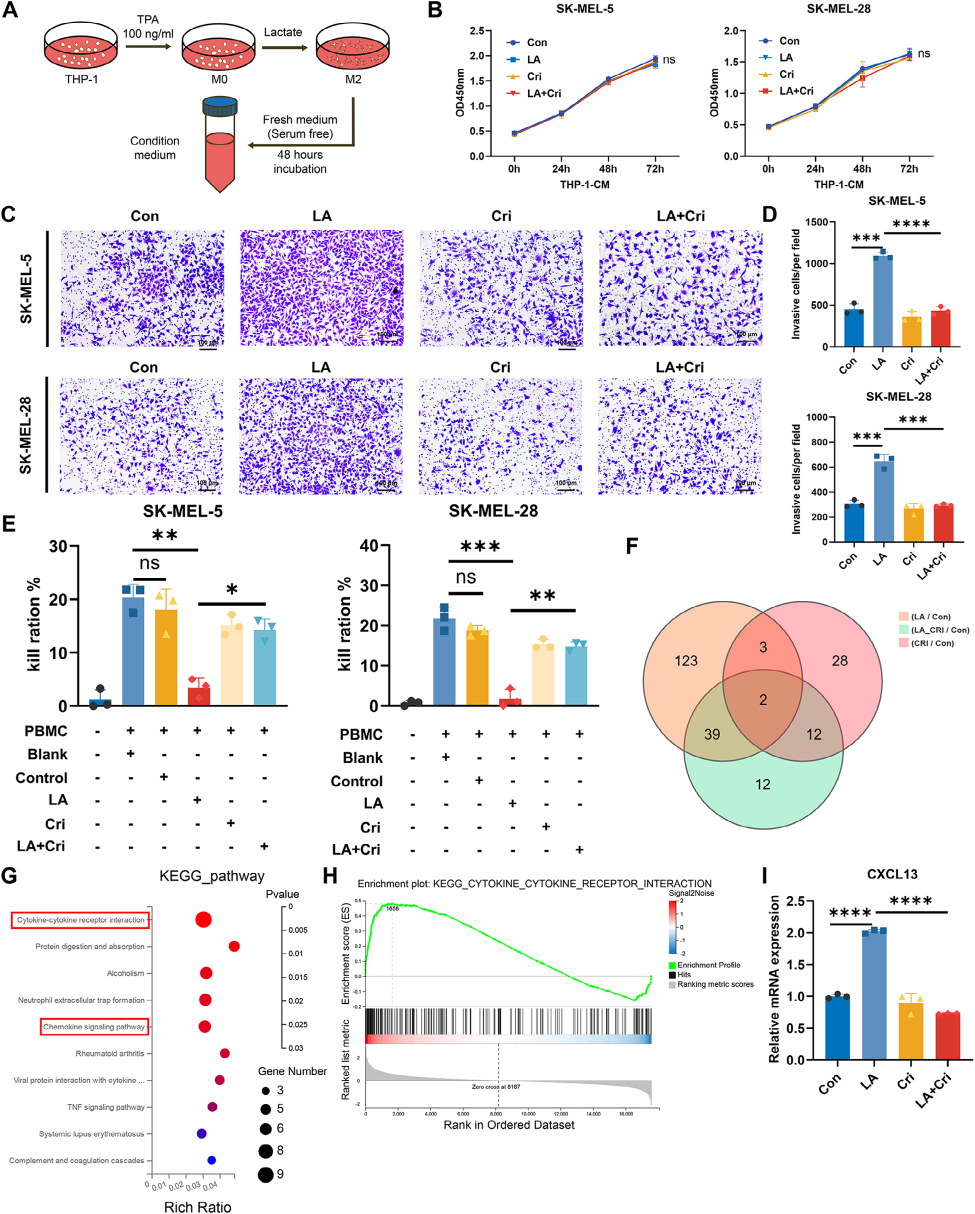
The impact of crizotinib on CXCL13 expression and tumor progression. (A) Schematic illustration detailing the collection of CM from THP‐1 cells, which were differentiated into M0‐phase macrophage‐like cells through stimulation with 100 ng/mL phorbol 12‐myristate 13‐acetate (TPA) for 24 h, followed by treatment with lactate for 24 h and subsequent incubation in serum‐free medium for an additional 48 h prior to CM collection. (B) Proliferation assays were performed on melanoma cells after exposure to macrophage CM (*n* = 3). (C and D) Transwell invasion assays were conducted to assess the changes in the invasive capacity of melanoma cells after treatment with macrophage CM, with accompanying statistical analyses (*n* = 3). (E) Melanoma cells were cocultured with PBMCs derived from human donors and treated with macrophage CM to evaluate the impact of the CM on cytotoxic activity (*n* = 3). (F) Transcriptomic sequencing was performed on macrophages after treatment, with a focus on lactate‐induced gene alterations, for further analysis. (G and H) KEGG pathway enrichment analysis was conducted on the DEGs. (I) Changes in the transcription of CXCL13 in response to lactate and crizotinib treatments were examined in THP‐1 cells (*n* = 3).

To investigate the influence of macrophages on melanoma, THP‐1 cells were polarized into M2‐like macrophages under previously described conditions, and the CM was collected (Figure 5A). We then assessed the effect of macrophage CM on melanoma cells. The results revealed that CM derived from lactate‐treated macrophages did not significantly affect tumor cell proliferation (Figure 5B) but significantly increased the invasiveness of melanoma cells (Figure 5C,D). This proinvasive effect was attenuated when macrophages were cotreated with crizotinib, indicating that lactate‐induced M2‐like macrophages can promote melanoma cell invasion. Given that crizotinib could enhance the effectiveness of PD‐1 treatment in mice with tumors, we hypothesized that changes in macrophage polarization status might affect the capacity of tumor‐killing immune cells. By coculturing peripheral blood mononuclear cells (PBMCs) with tumor cells in CM from different macrophages, we observed that lactate‐treated macrophage CM inhibited the PBMC‐mediated killing of melanoma cells (Figure 5E).

To investigate the underlying mechanism by which lactate‐induced macrophages affect immune cell‐mediated killing of tumor cells, transcriptomic sequencing was conducted, which revealed that lactate induced widespread changes in gene expression in macrophages (Figure 5F, Figure S4A,B). We selected genes whose expression was upregulated in the lactate‐treated group but whose expression was not significantly changed in the lactate‐ and crizotinib‐treated groups for further study. These genes represent lactate‐induced alterations that crizotinib could effectively intervene in. Gene ontology (GO) and Kyoto Encyclopedia of Genes and Genomes (KEGG) analyses of the differentially expressed genes (DEGs) revealed that lactate‐induced gene changes were involved mainly in cytokine‒cytokine receptor interactions, chemokine signaling, and cytokine activity pathways, with gene set enrichment analysis (GSEA) confirming these findings (Figure 5G,H, Figure S4C,D). Among these DEGs, significant changes were observed in the C‐X‐C motif chemokine ligand (CXCL) family, with CXCL13 showing the greatest fold change following crizotinib treatment (Figure S3E). Further validation through RT‒PCR confirmed that CXCL13 transcription in macrophages was upregulated under lactate induction and downregulated following crizotinib treatment, which was consistent with the transcriptomic sequencing results (Figure 5F, Figure S4F–H). Additionally, we observed a lactate concentration‐dependent change in CXCL13 expression (Figure S4I). These findings suggest that lactate induces macrophage polarization toward the M2 phenotype and may promote tumor progression and increase CXCL13 secretion.

### 2.6 CXCL13 Promotes the Invasion and Metastasis of Melanoma Cells and Enhances Their Immune Evasion

**FIGURE 6:**
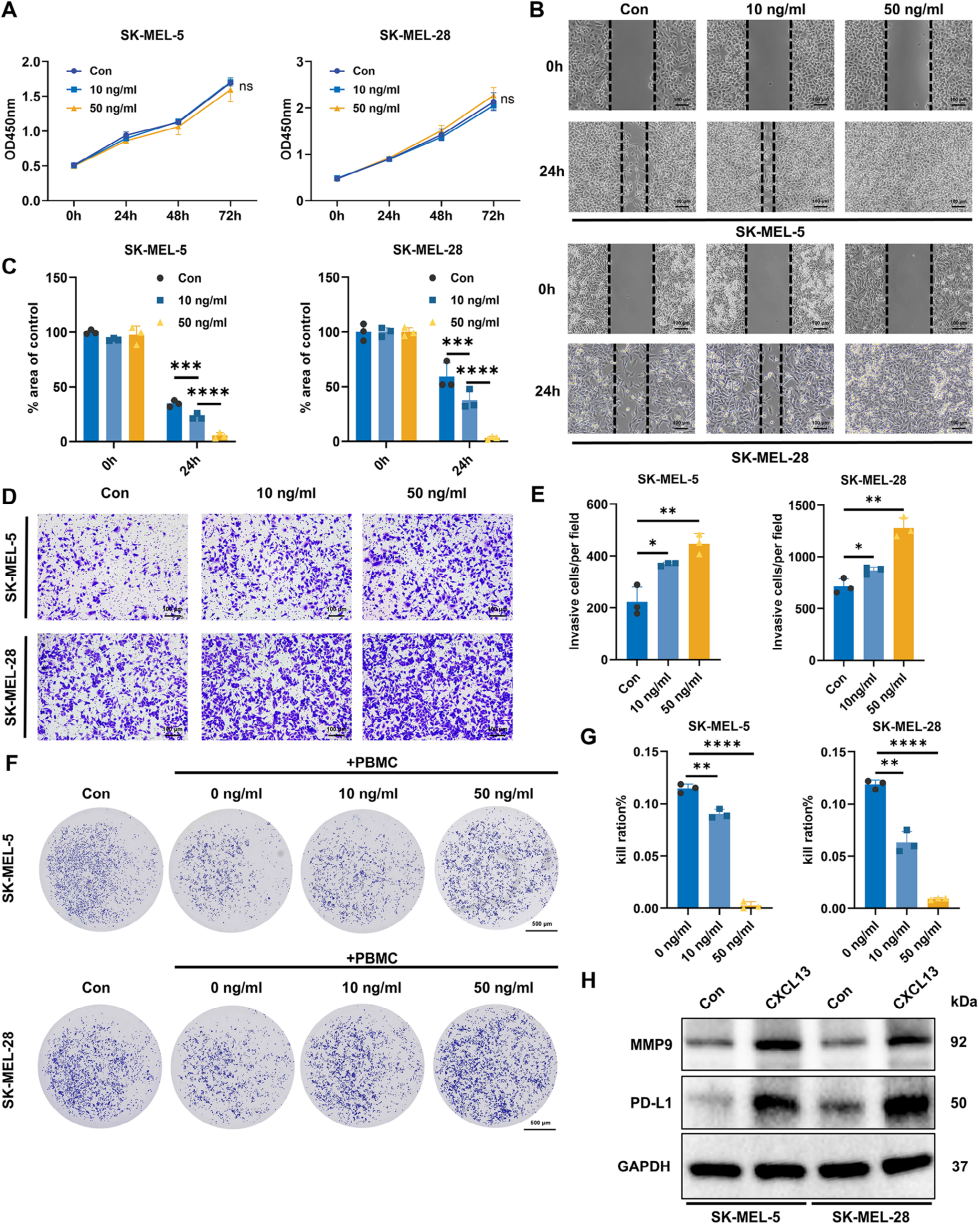
CXCL13 promotes melanoma progression by enhancing invasion and weakens PBMC‐mediated cytotoxicity. (A) SK‐MEL‐5 and SK‐MEL‐28 cell proliferation was assessed after CXCL13 treatment (*n* = 3). (B and C) Wound healing assays were conducted to assess the migration of melanoma cells treated with CXCL13 (*n* = 3). (D and E) Statistical analysis of the impact of CXCL13 on melanoma cell invasiveness (*n* = 3). (F and G) Melanoma cells were cocultured with PBMCs with and without CXCL13 (*n* = 3). (H) MMP9 and PD‐L1 expression was quantified in melanoma cells following treatment with 10 ng/mL CXCL13.

Given the cancer‐promoting role of M2 macrophages, we investigated the impact of CXCL13 on melanoma cells. CXCL13 treatment had no effect on melanoma cell proliferation (Figure 6A), but it markedly enhanced the invasion and metastatic potential of melanoma cells in a concentration‐dependent manner (Figure 6B–E). Furthermore, in vitro cytotoxicity assays revealed that the inhibitory effect of CXCL13 on melanoma cells in PBMCs could be reversed, with even 50 ng/mL CXCL13 completely reversing the PBMC‐mediated killing of melanoma cells (Figure 6F,G). To further understand how CXCL13 enhances the invasiveness and immune evasion capabilities of melanoma cells, we examined the protein levels in melanoma cells after CXCL13 treatment and observed a significant upregulation of matrix metallopeptidase 9 (MMP9) and programmed death‐ligand 1 (PD‐L1) (Figure 6H). These findings suggest that CXCL13 facilitates both the invasion and metastasis of melanoma cells, as well as their ability to evade immune surveillance, through the regulation of MMP9 and PD‐L1 expression.

### 2.7 Lactate Promotes CXCL13 Expression in Macrophages via H3K18la‐Mediated Histone Lactylation

**FIGURE 7:**
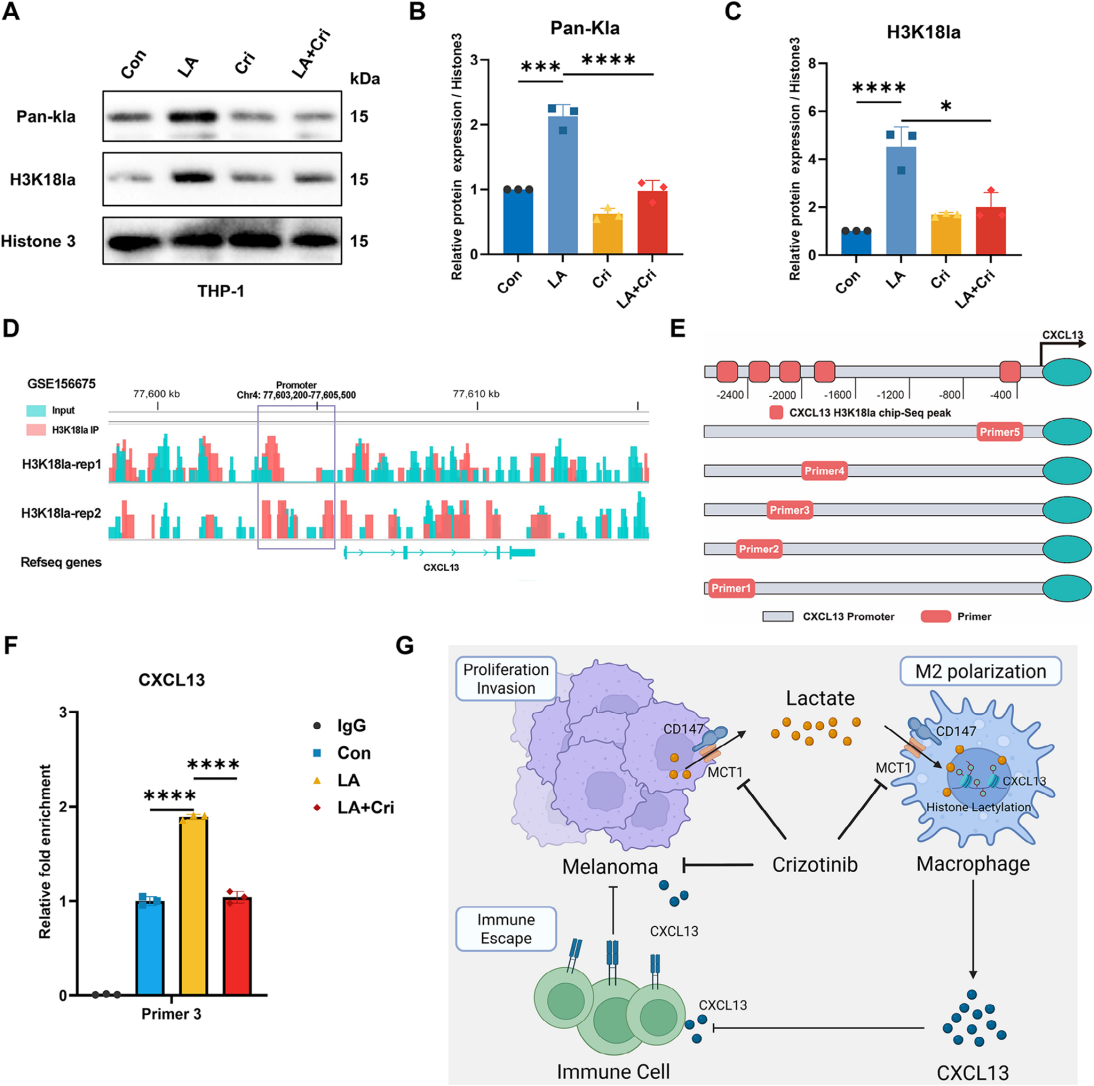
Macrophage histone lactylation promotes CXCL13 expression. (A) Histones were extracted from treated THP‐1 cells to assess overall lactylation and specifically H3K18 lactylation levels. (B and C) Statistical analysis of the lactylation levels. (D) ChIP‐seq peak analysis of CXCL13 was performed using the GSE156675 dataset. (E) A schematic representation of the CXCL13 primer design for H3K18la‐ChIP analysis. (F) ChIP analysis results and statistical data for primer 3 indicated that lactate enhances histone binding to the CXCL13 promoter region, whereas crizotinib inhibits this interaction (*n* = 3). (G) In melanoma cells, crizotinib impedes lactate transport by blocking the CD147–MCT1 interaction, reducing macrophage lactate uptake within the tumor microenvironment and thereby affecting CXCL13 secretion. This modulation influences melanoma cell invasion, metastasis, and immune evasion, with crizotinib attenuating lactate‐induced histone lactylation in macrophages.

Recent studies have revealed that lactate induces histone lactylation, an important epigenetic modification that influences chromatin structure, transcriptional regulation, and gene expression [11]. Based on these findings, we investigated whether histone lactylation was the mechanism underlying CXCL13 secretion by lactate‐treated macrophages. Immunoblots revealed a significant increase in histone lactylation in macrophages after lactate treatment, which was reversed after crizotinib treatment (Figure 7A,B). Histone lactylation occurs at multiple sites, of which histone H3 lysine 18 (H3K18) is the most widely studied, and is commonly associated with the regulation of gene expression. Thus, we focused on changes in the expression levels at H3K18. Consistent with the overall level of histone lactylation, lactate treatment upregulated H3K18 lactylation (H3K18la), which was also attenuated by crizotinib (Figure 7A,C).

To further investigate the impact of histone lactylation on CXCL13 expression, we utilized the GSE156675 dataset to predict potential histone binding sites within the CXCL13 promoter region and designed primers based on peak locations (Figure 7D,E). Chromatin immunoprecipitation (ChIP) and DNA gel electrophoresis confirmed that lactate significantly enhanced histone binding to the CXCL13 promoter in macrophages (Figure 7F, Figure S5). Together, these results suggest that lactate promotes CXCL13 expression in macrophages via histone lactylation of H3K18la.

## 3 Discussion

In recent years, monocarboxylate transporters (MCTs) have drawn significant attention due to their potential application in cancer treatment. Since tumor cells frequently rely on aerobic glycolysis for energy production, MCTs—particularly MCT1—play a crucial role in transporting monocarboxylates such as lactate across the cell membrane. As a result, MCT1 has emerged as a promising therapeutic target. Several selective small‐molecule inhibitors of MCT1, including BAY‐8002 and AZD3965, have been developed and evaluated in various tumor types [25, 26, 27]. Notably, AZD3965 has advanced to clinical trials [28]. Although MCT1 inhibitors show significant promise, most do not function through a direct disruption of protein‐protein interactions that are specifically targeted. Among various therapeutic strategies, targeting PPI has emerged as a promising approach due to its distinct advantages. Unlike the use of traditional small‐molecule drugs, the PPI strategy tends to have lower toxicity, as the interfaces of protein–protein interactions, effectively blocking crucial pathological signaling pathways. This high level of selectivity not only enhances therapeutic efficacy but also reduces the risk of side effects that often accompany nonspecific drug actions. For example, venetoclax (ABT‐199) precisely disrupts the interaction between B‐cell lymphoma 2 (BCL‐2) and BCL‐2‐like protein 11 (BIM), thereby inducing apoptosis in cancer cells. This specificity has resulted in successful applications for treating chronic lymphocytic leukemia (CLL) [29, 30] and acute myeloid leukemia (AML) [31], providing patients with a safer and more effective therapeutic options. Additionally, venetoclax has been shown to reduce the risk of thrombocytopenia in the context of breast cancer [22]. BET inhibitors, such as JQ1 and OTX015, represent a class of PPIs that modulate key gene expression by blocking the interaction between bromodomain and extraterminal domain (BET) proteins and acetylated histones. These inhibitors have shown broad therapeutic potential, demonstrating efficacy not only in hematological malignancies [32] but also in various solid tumor models, including prostate cancer [33], small cell lung cancer [34], and breast cancer [35]. These successful cases underscore that the application of PPI inhibitors in targeted therapy extends far beyond merely disrupting protein‒protein interactions and involves the precise regulation of complex gene networks. This precision in therapeutic intervention highlights the immense potential of PPI inhibitors in advancing the future of personalized medicine.

Lactate is a key by‐product of glycolysis and facilitates angiogenesis, thereby affecting tumor growth and metastasis [36, 37]. Additionally, lactate produced in the hypoxic TME, driven by hypoxia‐inducible factor 1‐alpha (HIF‐1α), significantly enhances ferroptosis resistance in solid tumors through a pH‐dependent mechanism [38]. Its impact on tumor behavior suggests a potential strategy for cancer therapy by targeting lactate metabolism. Given the critical role of lactate in tumor progression, our study aimed to identify specific inhibitors of the CD147–MCT1 complex, via a TR‐FRET‐based screening workflow. This approach successfully identified two promising compounds: crizotinib and axitinib. Of these, crizotinib was chosen as the lead candidate due to its ability to disrupt the CD147–MCT1 interaction, which was more pronounced than other inhibitors in IP assays. Originally developed as an ALK and ROS1 inhibitor for the treatment of non‐small cell lung cancer (NSCLC) [39], the newfound ability of crizotinib to inhibit CD147 and MCT1 extends its therapeutic potential into the realm of cancer metabolic reprogramming. Importantly, as an FDA‐approved drug, crizotinib possesses well‐characterized pharmacokinetic profiles and safety data, which facilitates its potential repurposing for the treatment of melanoma and other solid tumors. This “drug repositioning” strategy not only reduces the cost and time associated with de novo drug development but also enhances translational feasibility. Our findings demonstrate that crizotinib effectively inhibits melanoma progression by directly targeting CD147 and MCT1, thereby reducing lactate transport. These results suggest that crizotinib has great potential as a therapeutic agent for melanoma treatment.

In addition to its direct effects on tumor cells, lactate plays a crucial role in modulating the tumor immune microenvironment. Elevated lactate concentrations in the TME generate an acidic environment that facilitates immune evasion and dampens antitumor immune activity. These metabolic reprogramming events affect various immune cells, including T cells, the regulatory T cells (Tregs), myeloid‐derived suppressor cells (MDSCs), and macrophages [40, 41, 42]. Among these, macrophages—one of the most abundant immune cells in solid tumors—are especially sensitive to metabolic signals [43]. Increasing evidence indicates that lactate drives macrophages toward an immunosuppressive M2‐like phenotype by altering their function, reducing the secretion of proinflammatory cytokines (e.g., tumor necrosis factor‐alpha [TNF‐α] and interleukin‐6 [IL‐6]) [44] and increasing the production of anti‐inflammatory factors such as IL‐10 [45]. Moreover, TAMs have also been implicated in resistance to immunotherapy, and targeting M2 polarization has been reported to improve therapeutic efficacy. For example, inhibition of the nuclear factor kappa‐light‐chain‐enhancer of activated B cells (NF‐κB) pathway in M2 TAMs has been shown to reduce PD‐L1 expression, thereby enhancing the efficacy of PD‐1 blockade therapy [46]. Clinical studies in NSCLC patients have also demonstrated that lower CD163⁺ TAM infiltration correlates with better outcomes under immune checkpoint therapy [47]. In our study, crizotinib significantly inhibited M2 macrophage polarization induced by lactate and showed strong synergy with anti‐PD‐1 therapy, collectively enhancing antitumor immune responses. Further transcriptomic analysis revealed that CXCL13 transcription in macrophages was upregulated under lactate induction and downregulated following crizotinib treatment. As a chemokine, CXCL13 plays a significant role in cancer cell biology. Elevated CXCL13 expression, influenced by miR‐934 in colorectal cancer, enhances tumor invasiveness [48]. Additionally, CXCL13 can induce the expression of forkhead box P3 (Foxp3) in Tregs, which is crucial for suppressing tumor immunity [49]. Consistent with previous research, our findings indicate that CXCL13 facilitates both melanoma cell invasion and metastasis while also promoting immune evasion by modulating the expression of MMP9 and PD‐L1 in tumor cells. These findings underscore the multifaceted role of lactate in melanoma immunology.

Lactylation, recently identified as a posttranslational modification driven by lactate, is the “new favorite” epigenetic modification. Recent studies revealed that lactylation can impair the function of CD8^+^ T cells, increase Treg suppressive activity, and promote TAM‐mediated immune evasion, highlighting its systemic role in shaping an immunosuppressive TME [50, 51, 52]. These findings offer a new approach for overcoming immunosuppression by targeting lactylation in tumor treatment. A study by Zhao Y et al. [11] identified H3, H4, H2A, and H2B lactylation sites in HeLa cells and bone marrow‐derived macrophages (BMDMs), with species‐specific variations. Among these genes, H3K18 is the most extensively studied and is commonly linked to gene expression regulation. Research has shown that H3K18la upregulates the m6A reader protein YTH N6‐methyladenosine RNA binding protein 2 (YTHDF2), accelerating the degradation of period circadian regulator 1 (PER1) and tumor protein p53 (TP53) mRNAs and promoting malignancy in ocular melanoma [53]. In colorectal cancer, lactate produced by tumors induces H3K18 lactylation, inhibits retinoic acid receptor gamma (RARγ) transcription in macrophages, increases IL‐6 levels, and enhances tumor‐promoting functions [54]. Our study focused on the changes in expression levels at the H3K18 site because of its significant role. We found that crizotinib can suppress H3K18 lactylation, resulting in a notable decrease in histone binding at the CXCL13 promoter region. These findings reveal links between metabolism and epigenetic modifications in macrophages, offering a new approach to overcoming immunosuppression by targeting histone lactylation in melanoma treatment.

While this study provides novel insights into the role of crizotinib in regulating lactate metabolism and tumor immunosuppression via the CD147–MCT1 axis, several limitations should be acknowledged. First, although the disruption of the CD147–MCT1 interaction has been validated, some specific downstream mechanisms have not been directly examined in vivo, and its translational relevance to human clinical contexts also remains to be further established. Second, this study focused primarily on melanoma; whether the observed regulatory mechanisms extend to other tumor types has yet to be determined. Third, the tumor immune microenvironment is inherently complex and involves multiple interacting cell types. Although we observed clear effects on macrophage polarization and CD8⁺ T‐cell activity, a more comprehensive characterization of other immune components—such as dendritic cells, regulatory T cells, and MDSCs—will help clarify the broader immunological impact of crizotinib. Further investigations are warranted to elucidate the mechanism understanding this pathway and explore the potential of this pathway as a therapeutic target across a broader range of tumor settings.

## 4 Conclusions

Overall, the results from our study suggest that crizotinib can act as a CD147–MCT1 inhibitor and regulate lactate transport in both cancer cells and macrophages. The decrease in lactate induced by crizotinib significantly inhibits tumor proliferation, invasion, and metastasis. Additionally, crizotinib reduces lactate uptake in macrophages and hinders M2 polarization of macrophages, thereby enhancing the efficacy of PD‐1 checkpoint blockade immunotherapy in melanoma. Our results highlight the role of lactate, a key metabolite in the TME, in regulating immune cell metabolism to induce immunosuppression. This process is facilitated by the promotion of CXCL13 expression in macrophages via the histone lactylation of H3K18la. Considering the universality of lysine acylation, targeting lactylation presents novel opportunities to improve treatment outcomes and patient prognosis.

## 5 Methods

### 5.1 Cell Lines and Culture Conditions

SK‐MEL‐5, SK‐MEL‐28, THP‐1, and RAW264.7 cells were obtained from our laboratory. For culturing, SK‐MEL‐5, SK‐MEL‐28, and RAW264.7 cells were cultured in complete Dulbecco's modified Eagle medium (DMEM) (Gibco, C11995500BT, USA), while THP‐1 cells were grown in complete Roswell Park Memorial Institute (RPMI) 1640 (Gibco, C11875500BT, USA), with both media supplemented with 1% penicillin‐streptomycin and 10% fetal bovine serum (FBS) (ExCell Bio, 12J262, China). The cells were incubated at 37°C in a humidified environment with 5% CO₂.

### 5.2 TR‐FRET Assay

pcDNA3.2/V5‐DEST (Invitrogen, 12489019, USA) was used to construct Venus‐Flag‐CD147 and NLuc‐HA‐MCT1. HindIII and NheI restriction endonucleases were utilized to cleave the DNA strands, followed by ligation of the corresponding tag sequences. To express the CD147 and MCT1 proteins, 293T cells were transfected with Venus‐Flag‐CD147 and NLuc‐HA‐MCT1, followed by 48 h of culture. Subsequently, cell lysis was performed at 4°C using 0.5% NP‐40 lysis buffer (Beyotime, China). After high‐speed centrifugation, the supernatant was transferred to sterile ependorf tubes (EP tubes). Next, 384‐well plates (Corning, 3573, USA) containing anti‐Flag‐Tb (Cisbio, 61FG2TLF, France) and anti‐HA‐d2 (Cisbio, 610HADAF, France) TR‐FRET antibodies were prepared. The TR‐FRET antibody mixture was prepared at 2X concentration using a TR‐FRET buffer containing 50 mM Tris‐HCl, 20 mM NaCl, and 0.01% NP‐40, adjusted to pH 7.0. The library compounds (DMSO as a negative control), cell lysate, and 2X TR‐FRET antibody mixture (in a 1:1 volume ratio) were sequentially added to the 384‐well plate, followed by low‐speed centrifugation (1000 rpm, 2 min) and overnight incubation at 4°C. TR‐FRET signals were measured using a Cytation1 (BioTek, USA), and signal calculations were performed using the following formula: *F*_665 nm_/*F*_615 nm_ × 10^4^.

### 5.3 Pull‐Down Assay

Beads (Cytiva, 17043001, USA) were prepared and suspended in 1 mM HCl. The suspension was incubated in a rotary mixer for 5 min, followed by careful removal of the supernatant. This process was repeated three times. Finally, the supernatant was discarded. Crizotinib was prepared in a coupling buffer composed of 0.5 M NaCl and 0.1 M NaHCO_3_, adjusted to pH 8.3. Excess ligand was removed by washing with coupling buffer. The coupling solution containing the ligand was added to the beads and mixed thoroughly at 4°C overnight. The beads were transferred alternately into a solution of 1 M acetic acid (pH 4.0) and a second solution containing 0.5 M NaCl and 0.1 M Tris‐HCl (pH 8.0) and incubated in a rotary mixer for 5 min, and this process was repeated three times. Finally, the beads were rinsed in phosphate buffered saline (PBS), and after the supernatant was discarded, the beads were stored at 4°C in PBS with 0.01% sodium azide.

### 5.4 Histone Extraction and Western Blotting

Histones were prepared using a Histone Extraction Kit (Active Motif, 40028, USA). The protein samples were subsequently separated via 12% SDS‐PAGE. The membranes were blocked with 5% milk and incubated overnight at 4°C with either pan‐Kla (PTM BIO, PTM‐1401RM, China) or anti‐H3K18la (PTM BIO PTM‐1427RM, China). Immunoreactive signals were detected using the ChemiDoc XRS+ System (Bio‐Rad, USA).

### 5.5 Cell Proliferation Assays

The cells were seeded at a density of 2000 cells per well in 96‐well plates and treated with crizotinib or the control. Then 10 µL of CCK‐8 reagent (Selleck, B34302, USA) was added, followed by incubation at 37°C for 2 h. Absorbance was measured using a Cytation1 instrument (BioTek, USA), and the data were analyzed using Prism 8.

### 5.6 Wound‐healing and Transwell Assays

For the wound‐healing assay, cells were seeded into six‐well plates. A wound was generated with a sterile 10 µL pipette tip. The cells were washed twice with PBS before treatment under the indicated conditions. The cells were then subjected to the indicated conditions, and images of the wounded areas were captured at 0, 24, and 48 h.

For the Transwell assay, a 24‐well Transwell system (Corning, #3422, USA) was used. The cells were resuspended at 5 × 10^5^ cells/mL in serum‐free DMEM, and 100 µL of this suspension was added to each Matrigel‐coated insert. The lower chamber was filled with complete DMEM, and the plates were incubated at 37°C with 5% CO_2_. After incubation, noninvasive cells were removed, fixed with 4% formaldehyde, and stained with crystal violet. Image analysis was performed using ImageJ, and data and statistical analyses were conducted using Prism 8.

### 5.7 Mouse Models

Female C57BL/6 mice (6–8 weeks, Shanghai Laboratory Animals Center [SLAC], China) were used to establish the model. Approximately 5 × 10^5^ B16‐F10 cells were subcutaneously implanted into the right flanks of the mice. A YUMM1.7 melanoma model was established under the same conditions. When the tumor volume reached 50–100 mm^3^, the mice were randomly assigned to different groups. The mice were treated with crizotinib (25 mg/kg orally, MCE, HY‐50878A, USA) or/and an anti‐PD‐1 mAb (200 µg/mouse, Bio X Cell, BE0146, USA). Crizotinib was administered every 2 days, and PD‐1 was administered two times per week. The subcutaneous tumor size and animal weights were measured, and tumor volumes were calculated using the following formula: (length × width × height) × 0.5. The mice were euthanized, and the tumor tissue was harvested for analysis. This study was approved by the Ethics Committee of Xiangya Hospital (Central South University, China).

### 5.8 Flow Cytometry

Flow cytometry analysis was conducted on murine tumor tissues. Single‐cell suspensions were obtained by filtering through a 70‐µm cell strainer (Falcon, 352350, USA). For surface staining, the cells were incubated with fluorochrome‐conjugated antibodies specific to the target surface markers for approximately 30 min at 4°C in the dark. For intracellular staining, the cells were first restimulated for 30 min using the eBioscience Intracellular Fixation & Permeabilization Buffer Set (Invitrogen, USA), followed by incubation with antibodies against intracellular targets under the same conditions (4°C, in the dark, for 30 min). Analysis was performed using a FACS LSRFortessa instrument (BD Biosciences, USA).

### 5.9 Lactate Measurement

The lactate concentration was measured using a lactate colorimetric assay kit (Nanjing Jiancheng Bioengineering Institute, A019‐2, China). Lactate was converted to a chromogenic product and quantified spectrophotometrically at 530 nm.

### 5.10 RNA Sequencing and Data Processing

Total RNA was extracted and quality‐checked before being submitted to BGI Genomics (Shenzhen, China) for transcriptome sequencing. Library preparation and sequencing were performed using the DNBSEQ platform according to the manufacturer's protocols. Clean reads were then aligned to the human reference genome (Homo sapiens, GCF_000001405.39_GRCh38.p13, downloaded from NCBI) using HISAT2. The RNA‐seq data generated in this study have been deposited in the CNCB‐NGDC under accession number HRA011699.

### 5.11 Coculture of CXCL13 and PBMCs

PBMCs were isolated from healthy human blood samples and cocultured with tumor cells at a ratio of 5:1, with or without the addition of CXCL13. At the specified time points, the remaining cell populations were assessed to observe the impact of CXCL13 on the cytotoxic function of PBMCs.

### 5.12 ChIP Assays

ChIP assays were performed following the manufacturer's protocol (CST, 9003, USA). Cells were cross‐linked using 1% formaldehyde and subjected to sonication three times (20 s on, followed by 30 s of incubation on wet ice between each sonication). The sonicated chromatin was subsequently clarified via centrifugation. The cross‐linked chromatin was then diluted with 1X ChIP buffer for each reaction, and H3K18la antibodies (PTM BIO, PTM‐1427RM, China) were added to the supernatant. The control groups were treated with histone H3 (CST, 4620, USA) or rabbit IgG (CST, 2729, USA). Protein G magnetic beads were resuspended and added to each IP, followed by a 2‐h incubation at 4°C. The magnetic beads were separated, and the supernatant was removed. Chromatin was eluted through gentle vortexing (1200 rpm) for 30 min at 65°C. The cross‐links were reversed by treatment with proteinase K followed by incubation at 65°C for 2 h. The DNA was subsequently purified with spin columns. The PCR mixture was prepared following the manufacturer's guidelines and using standard parameters. The primer sequences used for ChIP‐qPCR are listed in Table S1.

### 5.13 Statistical Analysis

All in vitro experiments were repeated at least three times. Statistical significance was assessed via an unpaired two‐tailed Student's *t*‐test or one‐way analysis of variance (ANOVA), with GraphPad Prism 8 software. The error bars represent standard deviations, with statistical significance considered at *p < 0.05.

## Author Contributions

Z.Z. and X.Z. performed the experiments and contributed equally to the whole study. Z.Z., X.Z., S.Z., W.L., Y.G., S.X., C.P., and X.C. were responsible for providing the necessary reagents, conducting the experiments, and analyzing the collected data. Z.Z., X.Z., C.P., and X.C. analyzed the data and wrote the paper. All authors have read and approved the final manuscript. All the authors reviewed and accepted the contents of the article.

## Ethics Statement

All experiments were performed in accordance with the Ethics Committee of Xiangya Hospital (Approval ID number: 2023030354, Central South University, China).

## Conflicts of Interest

The authors declare no conflicts of interest.

## Supporting information




**Supporting File 1**: mco270286‐sup‐0001‐SuppMat.docx.

## Data Availability

The raw data supporting the findings of this study are available from the corresponding author upon reasonable request. The dataset supporting the conclusions of this article is included within its Supporting Information.
