# Supplementary material for: Crizotinib: A Novel Strategy to Reverse Immunosuppression in Melanoma by Targeting Lactate Transport
Source: MedComm (2020). 2025 Jul 21;6(8):e70286. doi: 10.1002/mco2.70286 (PMC12277666; doi:10.1002/mco2.70286)
Supplement: Supplementary file 1 — Supporting File 1: mco270286‐sup‐0001‐SuppMat.docx. [file MCO2-6-e70286-s001.docx]

**Crizotinib: A novel approach to reversing melanoma immunosuppression by targeting lactate transport**

Zhe Zhou^1,2,3,4,5#^, Xu Zhang^1,2,3,4,5#^, Susi Zhu^1,2,3,4,5^, Waner Liu^1,2,3,4,5^, Yeye Guo^1,2,3,4,5^, Siyu Xiong^1,2,3,4,5^, Cong Peng^1,2,3,4,5^*, Xiang Chen^1,2,3,4,5^*

1. The Department of Dermatology, Xiangya Hospital, Central South University, Changsha, Hunan, China
2. Furong Laboratory, Changsha, Hunan, China.
3. Hunan Key Laboratory of Skin Cancer and Psoriasis, Xiangya Hospital, Central South University, Changsha, Hunan, China
4. Hunan Engineering Research Center of Skin Health and Disease, Xiangya Hospital, Central South University, Changsha, Hunan, China
5. National Clinical Research Center for Geriatric Disorders, Xiangya Hospital, Central South University, Changsha, Hunan, China.

# Z.Z. and X.Z. contributed equally to this work.

* **Corresponding author**: Cong Peng, Department of Dermatology, Xiangya Hospital, Central South University, Changsha, Hunan, China, 410008. Email: pengcongxy@csu.edu.cn (Cong Peng)

* **Corresponding author**: Xiang Chen, Department of Dermatology, Xiangya Hospital, Central South University, Changsha, Hunan, China, 410008. Email: chenxiangck@csu.edu.cn (Xiang Chen)

**Table S1. ChIP-qPCR primers**

| **Gene** | **Sequences (5’-3’)** |
| --- | --- |
| CXCL13-site 1-Forward | ACTTGGGTCTCAAGATCATATCACA |
| CXCL13-site 1-Reverse | TGGTAGGCAATAACATTCCTGT |
| CXCL13-site 2-Forward | GCTTGTACTGGCAAAGCCAT |
| CXCL13-site 2-Reverse | TGACTCACAGCCACAGACAC |
| CXCL13-site 3-Forward | TGGGTTTTGGAGTGGAAGTTAGT |
| CXCL13-site 3-Reverse | CTGCTCCATTGCTTCTACATTTCT |
| CXCL13-site 4-Forward | GTATTTGGCCGAACCCTCTAC |
| CXCL13-site 4-Reverse | CTGGCAATGACTTTGGACAGGT |
| CXCL13-site 5-Forward | CACCTACCAAAGGCCGGAAT |
| CXCL13-site 5-Reverse | CATGACATTGGGGACTCCGT |


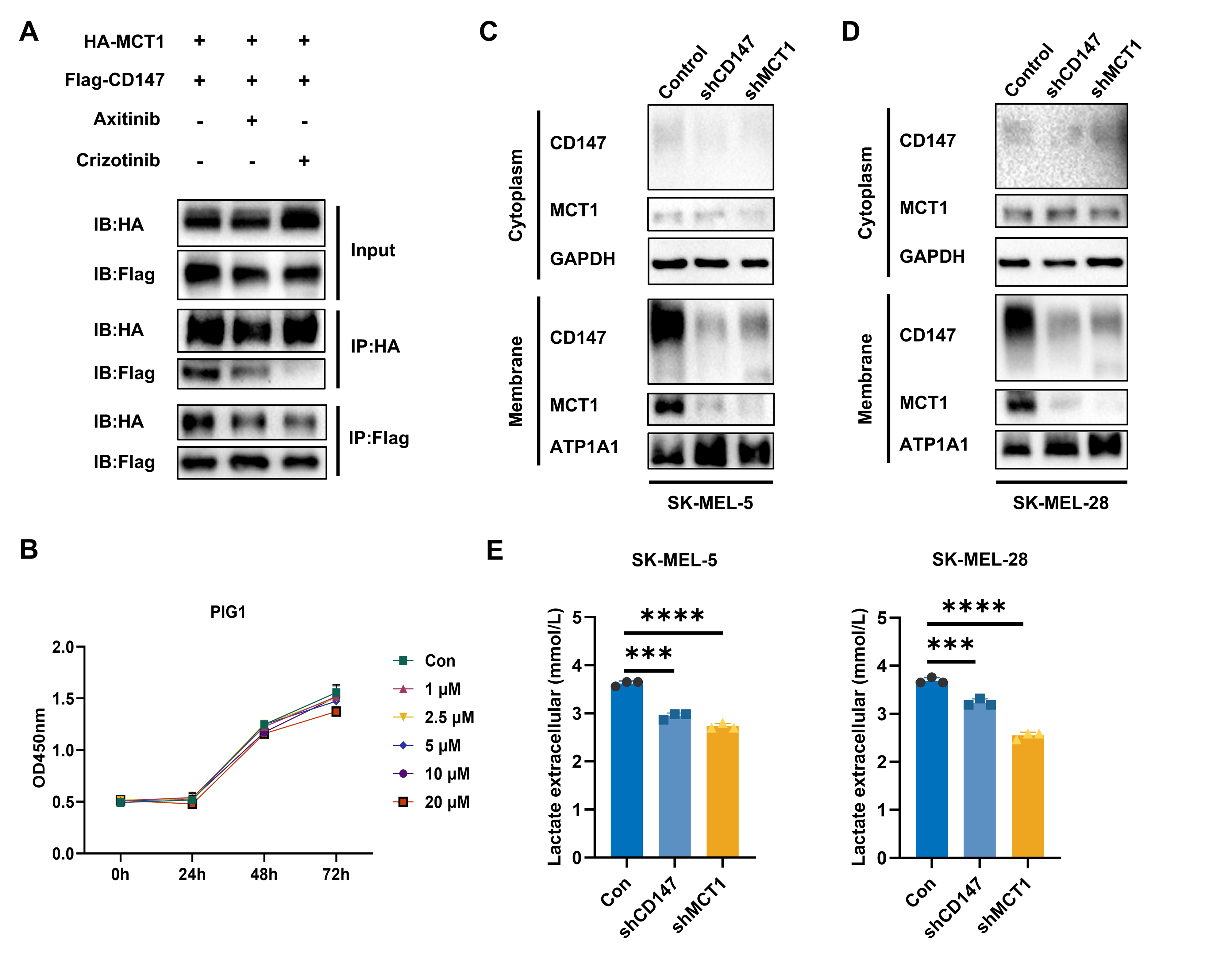


**Figure S1.** **Pharmacological and Genetic Disruption of CD147-MCT1 Impairs Lactate Transport**

**(A)** HA-MCT1 and Flag-CD147 were co-overexpressed in 293T cells, which were then treated with axitinib or crizotinib. The impact of these inhibitors on the CD147-MCT1 interaction was evaluated via an immunoprecipitation assay. Among these two inhibitors, crizotinib demonstrated a greater capacity to disrupt the CD147-MCT1 interaction. **(B)** The inhibitory effect of crizotinib on PIG1 (n = 3). **(C-D)** Cytoplasmic and membrane protein fractions were isolated from melanoma cells to determine the effects of CD147 knockdown on MCT1 localization. **(E)** Lactate concentrations in the supernatant were quantified after the knockdown of CD147 or MCT1 (n = 3).


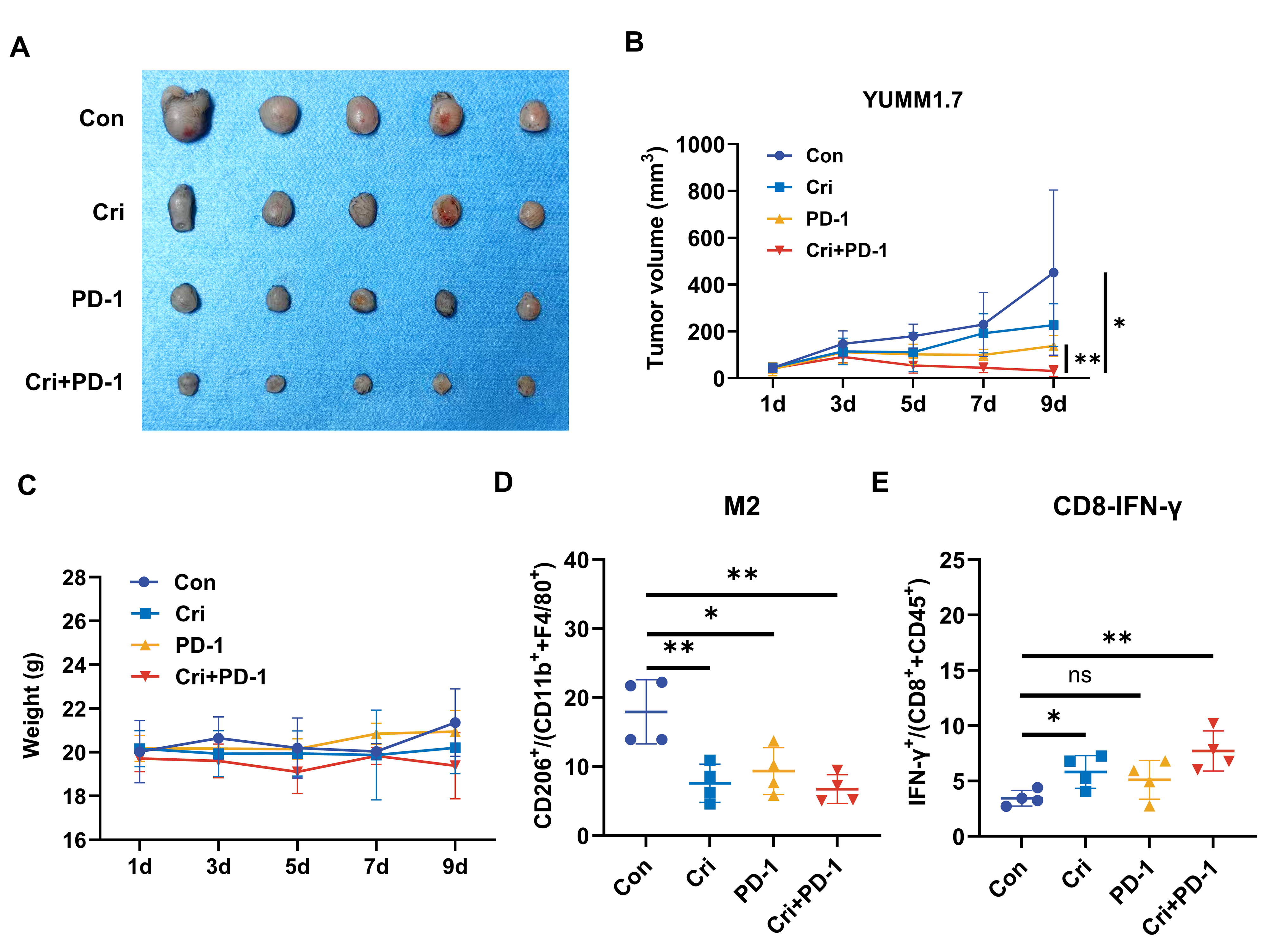


**Figure S2. Crizotinib Enhances PD-1 Blockade in the YUMM1.7 Melanoma Model**

**(A)** Representative images of tumors collected from mice (n = 5 mice per group). **(B)** Tumor volume measurements over the course of treatment; the data are shown as the means ± SDs. **(C)** Body weight monitoring throughout treatment. **(D)** Quantification of tumor-infiltrating M2 macrophages (CD206⁺CD11b⁺F4/80⁺, n = 4 mice per group). **(E)** Proportion of IFN-γ⁺ CD8⁺ T cells (CD8⁺CD45⁺).


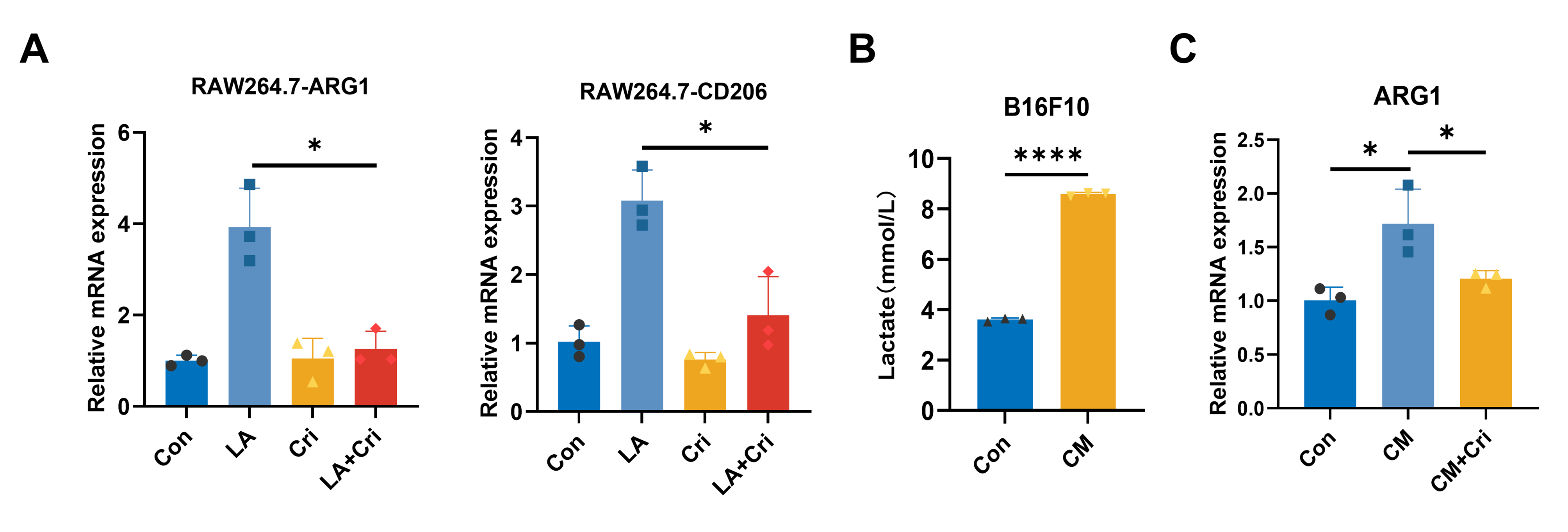


**Figure S3. Crizotinib Inhibits Lactate Uptake and M2 Polarization in RAW264.7 Cells**

**(A)** The transcription of M2 markers was analyzed in RAW264.7 cells treated with lactate, with and without crizotinib (n = 3). **(B-C)** Conditioned medium from B16F10 cells was collected, and the lactate level in the supernatant was measured. The macrophages were subsequently exposed to this medium, and the transcriptional changes in M1/M2 markers were assessed (n = 3).


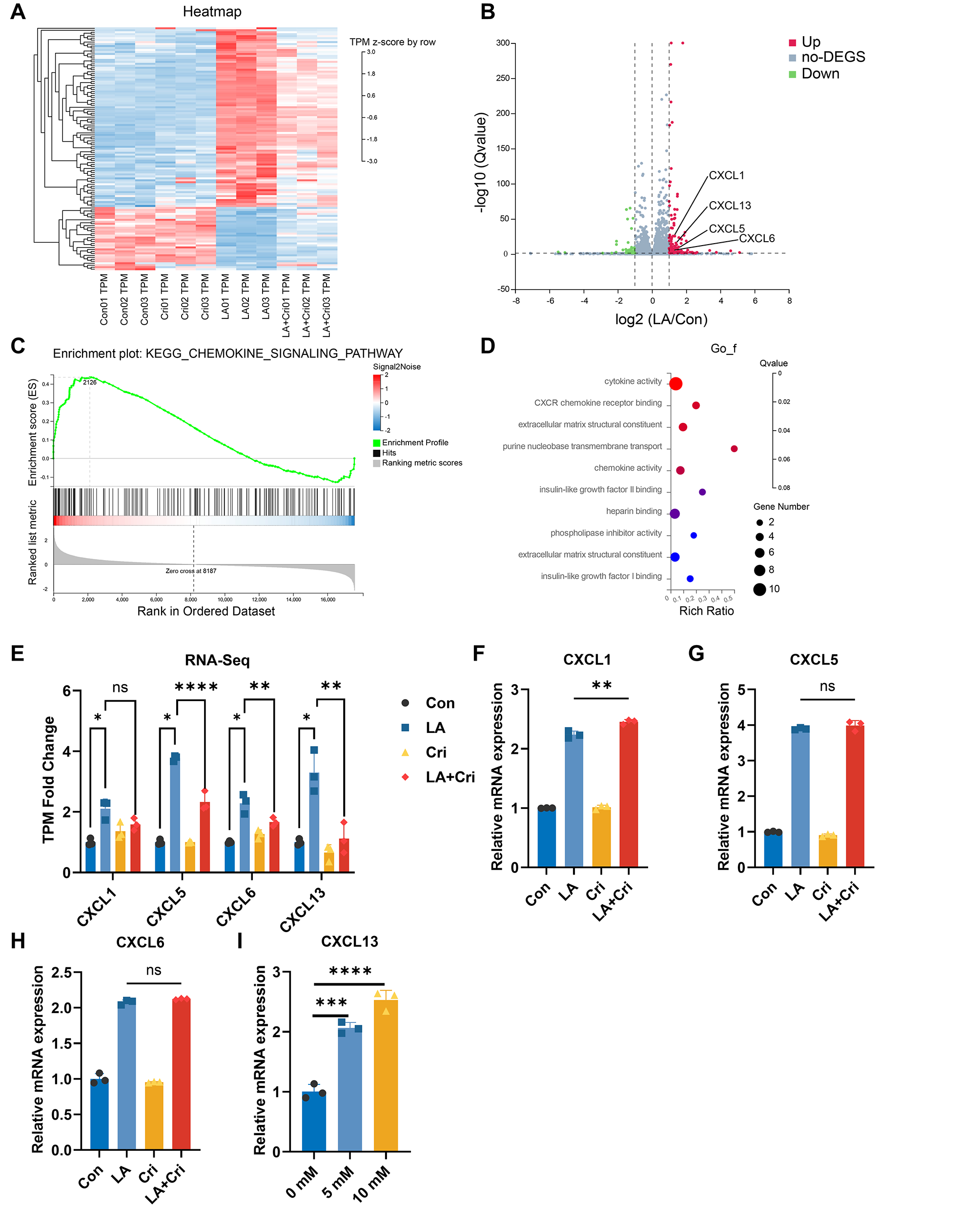


**Figure S4. Crizotinib Reverses Lactate-Induced Inflammatory and Chemokine Signatures in Macrophages**

**(A)** Heatmap showing the transcriptomic profiles across different experimental groups. Each group included three biological replicates (n = 3). **(B)** Volcano plot highlighting genes whose expression was uniquely significantly upregulated or downregulated. **(C)** KEGG pathway analysis revealed that the DEGs are involved in cytokine signaling pathways. **(D)** GO-F enrichment analysis showing the functional categories of the DEGs. **(E)** Expression levels (TPM) of the key chemokines CXCL1, CXCL5, CXCL6 and CXCL13 (n = 3). **(F-H)** The expression of CXCL1, CXCL5, and CXCL6 was further validated by RT‒PCR in THP-1 cells treated with lactate and crizotinib (n = 3). **(I)** The expression of CXCL13 in macrophages was measured at different lactate concentrations and revealed a clear dose-dependent response (n = 3).


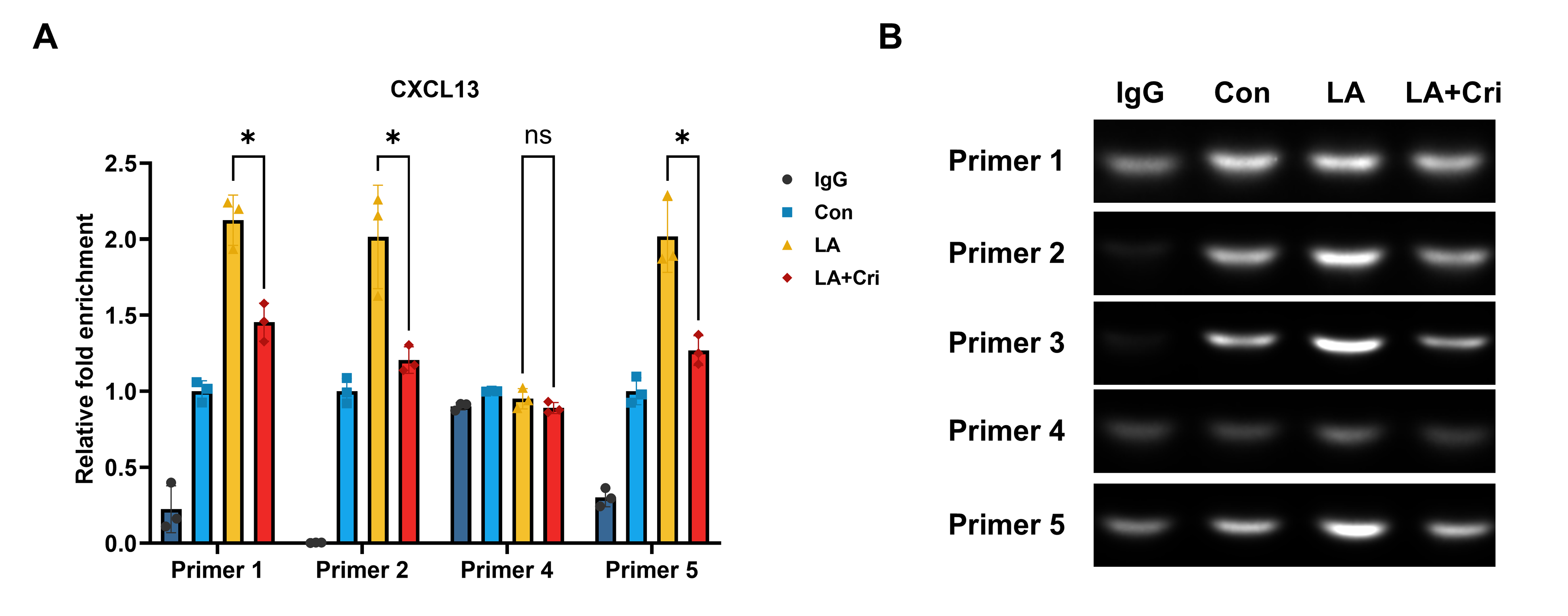


**Figure S5. ChIP Analysis of H3K18la Modification**

**(A)** ChIP results showing the relative fold enrichment of H3K18la at the genomic regions amplified by Primers 1, 2, 4, and 5 (n = 3). **(B)** Gel electrophoresis of the PCR products from the ChIP assay, corresponding to primers 1-5.
